# Percolation and tortuosity in heart-like cells

**DOI:** 10.1038/s41598-021-90892-2

**Published:** 2021-06-01

**Authors:** R. Rabinovitch, Y. Biton, D. Braunstein, I. Aviram, R. Thieberger, A. Rabinovitch

**Affiliations:** 1 Makif YudAlef, Rishon Lezion, Israel; 2 Physics Department, Ben-Gurion University, Beer-Sheva, Israel; 3 Physics Department, Sami Shamoon College of Engineering, Beer-Sheva, Israel

**Keywords:** Cardiovascular biology, Computational biophysics, Cardiovascular biology

## Abstract

In the last several years, quite a few papers on the joint question of transport, tortuosity and percolation have appeared in the literature, dealing with passage of miscellaneous liquids or electrical currents in different media. However, these methods have not been applied to the passage of *action potential* in heart fibrosis (HF), which is crucial for problems of heart arrhythmia, especially of atrial tachycardia and fibrillation. In this work we address the HF problem from these aspects. A cellular automaton model is used to analyze percolation and transport of a distributed-fibrosis inflicted heart-like tissue. Although based on a rather simple mathematical model, it leads to several important outcomes: (1) It is shown that, for a single wave front (as the one emanated by the heart's sinus node), the percolation of heart-like matrices is exactly similar to the forest fire case. (2) It is shown that, on the average, the shape of the transport (a question not dealt with in relation to forest fire, and deals with the delay of action potential when passing a fibrotic tissue) behaves like a Gaussian. (3) Moreover, it is shown that close to the percolation threshold the parameters of this Gaussian behave in a critical way. From the physical point of view, these three results are an important contribution to the general percolation investigation. The relevance of our results to cardiological issues, specifically to the question of *reentry initiation*, are discussed and it is shown that: (A) Without an ectopic source and under a mere sinus node operation, *no arrhythmia* is generated, and (B) A sufficiently high refractory period could prevent some reentry mechanisms, even in partially fibrotic heart tissue.

## Introduction

The combination of percolation, transport and tortuosity is proving to be of great importance in many scientific areas.

Percolation is a well-established mathematical field (See e.g. review^1^), which has been applied to quite a few physical, biological and medical domains.

Critical exponents are numbers describing systems' behavior near second order (continuous) phase transitions in thermodynamic systems and near percolation transitions. They are considered to be independent of the actual or specific system, and to depend only on the systems' intrinsic character such as the system's dimensions and the nature of interactions between its particles. Percolation problems deal with the clustering of identical substances randomly and uniformly distributed in space with an occupation probability *p*, hindering passage through the system. For an infinite system, there exists a certain threshold probability, *p*_*c*_, above which passage through the system is completely blocked. For finite systems the transition is gradual. Near *p*_*c*_, some physical properties do not depend on the actual substances nor on the clustering structures. These properties behave, asymptotically, only as a power law of the distance from *p*_*c*_, i.e., they are proportional to $\left( {p - p_{c} } \right)^{\beta }$, where β is a constant (the critical exponent for this property).

One of the directions in which percolation theory has evolved is the so-called forest fire model (FFM)^2, 3^, a part of a general "directed percolation" (DP) models, where the percolation can only move in a single forward direction and backwards movement is prohibited, such as for fire propagation. Electrical and thermal passage through different media have also been investigated by percolation methods^4^. For a review see Ref.^5^. Tortuosity and transport issues (not dealt with in FFM) are considered in combination with percolation for different materials and passage avenues. For a critical review see Ref.^6^.

Electrical movement in neurons, in the heart and brain tissues, is what we want to call "semi directed percolation" (SDP). Here, once a cell has operated, it passes into a "refractory period" in which it is prevented from reactivation. However, in contrast to FFM, the refractory period persists only for a limited time, allowing movement in the opposite direction. SDP therefore seems to be a *different type of percolating systems*.

Percolation and transport in such SDP media may have important implications in cardiology^7–10^, where malfunction in action potential movement can lead to tachycardia and fibrillation, and in brain investigations^11^. The transport and delay of arrival of action potential through fibrotic tissues are of major importance in understanding the initiation of heart arrhythmias (for an expansion of this issue see the "Discussion" section). However, although simple percolation (see e.g. ^12^. for a recent approach to the subject) and diffuse ^13^and anisotropic conduction^14^ through heart tissues has been considered, there has been no systematic investigation of the combined issue of percolation, transport and tortuosity for the SDP problem. Note that "tortuosity" in the heart tissue is referred to as zig zag movement based on the seminal^15^ study. We start such systematic investigation in this paper. We restrict ourselves here to heart-like cells since in the brain realm the system is further complicated by the existence of two kinds of cells there, excitatory and inhibitory ones. We consider here only excitatory type of cells.

Here we treat "forest fire" and heart-like cellular automata models to understand site percolation of these systems under increasing percentages of non-operating cells. For the heart-like SDP we only use cases where the refractory period is equal or larger than the active one. The case of shorter refractory periods will be dealt with in future publications.

It turns out that, in the sense of percolation, both systems, the FFM and the SDP kind examined here, behave *likewise*, which could have been somewhat surprising. In contrast to the FFM case, however, for the heart case the transport of the process is of major importance, namely, how many end-cells are activated (and the delay and locations of the arriving action potentials. Therefore, besides percolation itself, two additional features, the fraction of the starting "objects" that reaches the other side of the percolating area (percolation throughput or transport^16^) and the delay time in this fraction arrival there (average time of arrival, related to the tortuosity^6, 17^ or zigzag^13, 15^ of the AP route) are treated here. The throughput or transport and the delay (related to the tortuosity of the routes it took) in its arrival at the other side and the spread in arrival times are measured by the Gaussian parameters of the average fraction. These questions of transport are prevalent in hydrological and other geological domains (e.g.^6^) and there is a vast amount of literature devoted to them, but not so for the heart problem. Note, however, that transport here is *different* from hydraulic or electrical conductivity, in which movement occurs under Darcy's law or Ohm's law (see e.g. Ref.^10^), since there is no voltage or pressure difference which drives the AP across the heart. AP movement is nonlinear and is simulated here only by the influence of one cell on its immediate neighbors.

In the present percolation problem, we are also going to look for physical properties which behave critically near the percolation threshold. Based on Ref 10, the two obvious candidates are the tortuosity and the throughput. Indeed, as we shall see, near enough to the percolation point, these properties do behave in such a way.

## Model

The model we use is a simple 2D square cellular automata grid of n × n cells. Time is discrete and measured by generations (or time units).

Each cell at each generation can assume several characteristics denoted by digital numbers from 0 to 2 (and 9, see below). A cell, which is at mode 1, is operating, namely containing action potential (AP). The label 0 denotes a "waiting" cell, a cell that can be excited into an operating one (see below). Label 2 denotes cells in their refractory period in which no stimulation by neighboring cells can induce them to operate. The label can be increased to 3, 4 etc. to relate to the length of the refractory period (in generations) which depends on the last number used for the particular "trial".

Only nearest neighbors of a cell, i.e., the four cells closest to the specific cell, and not next nearest ones (those diagonally bordering it) are considered here as "cell neighbors" which can transfer it to be active.

In the "diagonal" percolation case, treated here as well, the rule is different, i.e., only the "upper" two diagonally bordering cells of a specific cell can influence it.

Label transitions from one generation to the next is done for all cells according to the following rules:

- A cell labelled 1, is transferred to label 2 (if present in this trial)
- A cell labelled 2, passes over to label 3 (if present in this trial), 3 goes to 4 etc.
- A cell with the final label of refractoriness for this trial, changes to 0 in the next generation. For example, for equal operating (1) and refractory (2) periods, only these labels exist and the system follows the rule: $1 \to 2 \to 0$.
- A cell labelled 0, transfers to 1 in the following generation if one or more of its "cell neighbors" was operative, i.e., labelled 1 at the present generation.
- For the FFM case, a cell whose label is 1, transfers to 9 (inoperative) in the following generation.

The percolation in the system is investigated by rendering, at the beginning of each trial, a specific percentage, *q*, of cells to be inoperative, labelled 9 (site percolation). These are randomly spread among all grid cells, excluding the first and last rows of the grid. The percolation percentage is $p = 100 - q$.

A "run" consists of starting the system (matrix) thus built, by a wave of AP, created as elucidated below, and running the system for enough generations (6 times the matrix size) so that all "residual" AP's can be collected at the final row. A "trial" consists of 40 runs, each performed for different random starting systems. Trials were performed for each *p* between 0 and 100 (*q* between 100 and 0).

At the start of each trial, all cells except those having a label 9 and those in the first row, are labelled 0. Cells of the first row are turned operative (labelled 1). This arrangement insures an AP wave through the system. In this work, only a single front of AP wave is treated. In this way, no active reentry mechanism is developed in the heart like system (see "Discussion"). Two consecutive waves, repeated stimulation in this framework and waves approaching from orthogonal directions will be discussed in future investigations.

We treat two questions here.

The first is a comparison of the percolation threshold of an SDP and of three others: a forest fire one, a different unidirectional system and a system devoid of refractory. The second is the transport and tortuosity of a system in which refractory time is equal to operative time (1 → 2 → 0), of different sizes and under increasing numbers of inoperative cells.

The first problem includes four systems of 100 × 100 cells are analyzed. These are: (1) A system with no refractory (1 → 0). (2) A system with a refractory period of a single time unit (1 → 2 → 0) (3) A system similar to "forest fire" model (1 → 9). (4) A DP in which a cell transfers from 0 to 1 only if one, or both, of its two upper diagonal neighbors is operative. Forty matrices of each system are treated, and the number of trials, in which at least one active cell survives, are counted and drawn as a function of *p*.

For the second issue, diverse sizes of (1 → 2 → 0) systems are analyzed, each with a number of different random *q*'s and for a single starting wave of AP.

**Figure 1:**
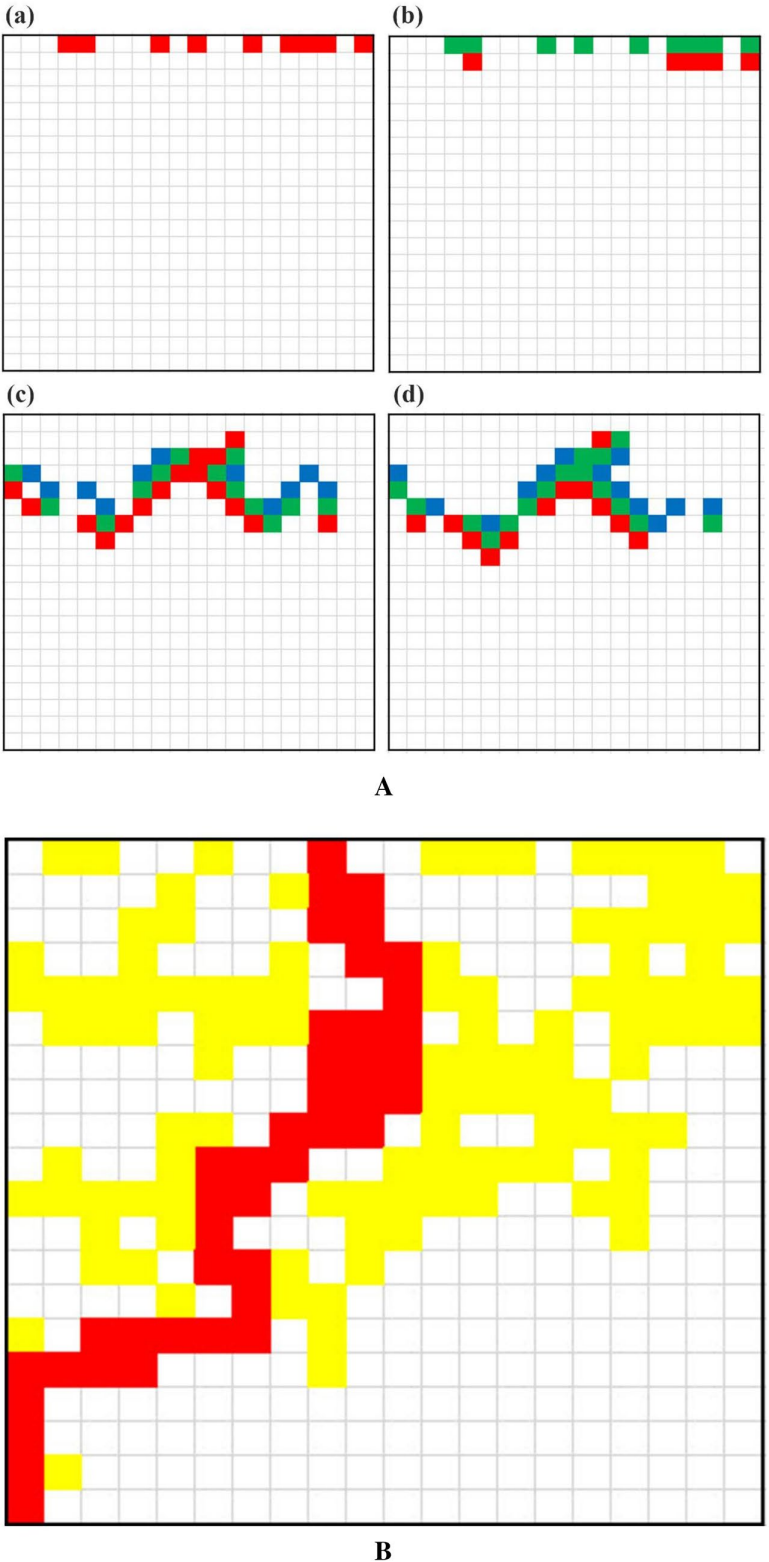
(**A**) Several generations of a typical case: Matrix 20 × 20, *p* = 60%. Generations presented: (a)-1, (b)-2, (c)-8 and (d)-9. Cells of type 1 are in red; those of type 2 in green; and those of type 3 in blue. (**B**) Exemplary active-cells through-path (in red) near percolation. Matrix 20 × 20, *p* = 60%. Other active cells during process are in yellow.

Several generations of a typical case are presented in Fig. 1A, where the complex development of the AP activation through the system is manifested. Numbers of active cells arriving at the last row (also called "arriving AP") are counted as a function of time units for a long time (6 times system's size). Figure 1B depicts an exemplary route of such active cells through the system very near the percolation threshold. The total number of such cells is called "throughput' while the normalized average delay of arrival is the "tortuosity". If the system is devoid of obstacles (zero number of 9-cells), all active cells would arrive there exactly after a number of time units equivalent to the system's size. In this case throughput is maximal and tortuosity is one. With obstacles, some activity is lost, so throughput is diminished and active cells are delayed on their route to the end row, so tortuosity is increased. The trial average of 40 runs of numbers of active cells arriving at the last row as a function of time (units) is approximated by a Gaussian distribution. Near percolation, critical exponents for the parameters of this Gaussian are computed.

## Results

### Percolation threshold

Perhaps surprisingly (see "Discussion"), both percolation results for an SDP system with a long refractory period (not shown) and those for the FFM turned out to be *exactly the same* as those of an SDP with equal active and refractory periods (1 → 2 → 0). Moreover, when analyzing a matrix with the *same* randomly distributed inoperative cells, results of the *complete spatiotemporal development* of active cells for the FFM and for the SDP systems turned out to be the same. Therefore, no regular critical exponents for the 1 → 2 → 0 will be presented case, as they are also identical to those of the FFM (1 → 9).

**Figure 2:**
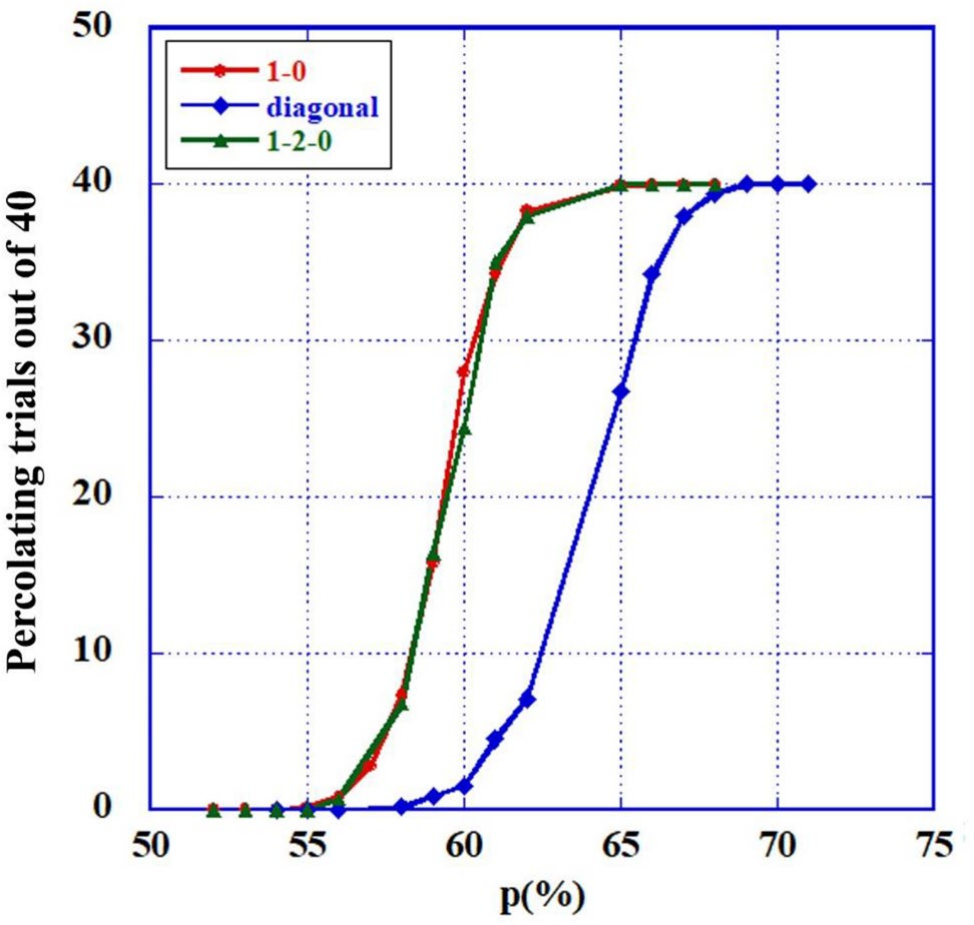
(Colors on line) Percolation curves for the four cases. Red (circles)—the **1 → 0** case. Green (triangles)—the **1 → 2 → 0** and the FFM cases. Blue (squares)—the "**diagonal**" unidirectional case.

Figure 2 represents the percolation results of these cases. All percolations occur at around $p = 59.3$. The likeness of the percolation graphs of the 1 → 2 → 0 case and that of the 1 → 0 case (no refractory period) is probably unexpected, since the results for each run, on matrices of the two cases with the *same* randomly distributed inoperative cells, are *vastly* different. This difference could clearly have been predicted, since for such runs, the amount of active cells reaching the end row is much higher for the second case.

### Throughput and delay (transport and tortuosity) for the SDP, 1 → 2 → 0

**Figure 3:**
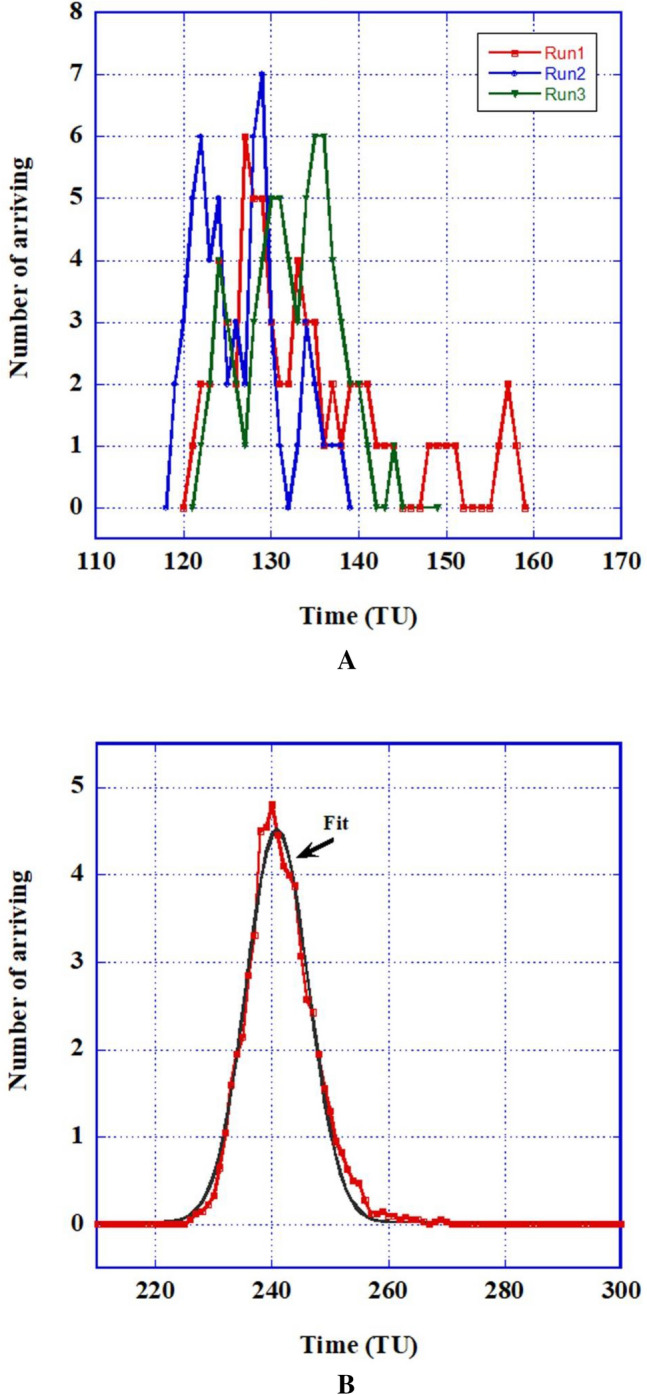
(Colors on line) Output (arriving active cells at the last row) for *p* = 70%. (**A**) Three individual runs of 100 × 100 matrices with different random distributions of inactive cells. (**B**) Average output of forty 200 × 200 matrices and the Gaussian fit.

Results show that, indeed, for the SDP percolation, both the throughput of the arriving AP and its delay are altered with *p*. Figure 3a shows numbers of output AP's as functions of the generations post-traversing the percolation matrix of three examples of individual runs, and Fig. 3B depicts the average throughput of a trial of 40 runs, all for a specific inoperative cells fraction of 30 (*p* = 70).

As can be seen from Fig. 3, individual trials are widely different from each other, especially near percolation threshold. Considering the average of many trials, a Gaussian behavior emerges. The slight right-tendency is neglected. This average may be fitted by a normal distribution curve, namely: $$n = A\exp \left[ { - \left( {{\raise0.7ex\hbox{${\left( {x - x_{0} } \right)^{2} }$} \!\mathord{\left/ {\vphantom {{\left( {x - x_{0} } \right)^{2} } {2\sigma^{2} }}}\right.\kern-\nulldelimiterspace} \!\lower0.7ex\hbox{${2\sigma^{2} }$}}} \right)} \right],$$ whose parameters, $$A$$, $$x_{0}$$ and $$\sigma$$ provide information related to the final amount, the average delay of its arrival and the average spread of the latter. Here, $$x_{0}$$ is the time when $$n$$ is at its maximum height, $$A$$, and $$\sigma$$ is the Gaussian width in time units.

Table 1 provides the results of these parameters and of 2.507 $x_{0} \sigma$, which is a measure of the average throughput. In our case, $x_{0}$ is measured in generations (time units) or in units of cell length. Note that here, these two parameters, namely the time (measured in generations) and the length of the route taken (measured in cell lengths), have the *same value*. This is unlike the hydrologic cases where these two are usually different. The reason for their equality here is that the AP can move down the region only one cell per generation. The minimum number of generations necessary for a starting AP to reach the opposite side of the investigated region is the region's length in unit cells, *L*. The average length of the route, $l_{0}$, taken by the AP to move from the initial position to the final one, is $x_{0} + L$. Thus, the geometrical tortuosity (see e.g. Ref.^10^), is:

1 $$\tau = \frac{{\left\langle {l_{0} } \right\rangle }}{L} = {\raise0.7ex\hbox{${x_{0} }$} \!\mathord{\left/ {\vphantom {{x_{0} } L}}\right.\kern-\nulldelimiterspace} \!\lower0.7ex\hbox{$L$}} + 1$$

**Table 1:** Gaussian parameters ($x_{0}$, $\sigma$, *A* and $AT = 2.507A\sigma$) of the average output of active (AP) cells as functions of *p* for L = 30, 70, 100 and 200.

$L$	$p$	$x_{0}$	$\sigma$	$2.507{\kern 1pt} a\sigma$
30	95	30.02	0.8436	28.28
	90	31.061	1.115	26.25
	85	32.22	1.394	24.46
	80	33.66	1.73	22.23
	75	35.46	2.27	20.34
	70	38.05	3.176	20.34
	65	41.32	4.497	11.85
70	95	71.28	1.12	64.41
	90	73.67	1.52	61.16
	85	76.4	1.9	51.15
	80	76.4	2.42	51.15
	75	84.18	3.24	47.92
	70	90.35	4.76	41.76
	65	100.7	7.85	30.70
	64	102.37	8.48	27.21
	63	106.47	10.81	24.39
100	95	102.27	1.22	95.45
	90	105.69	1.636	89.82
	85	109.63	2.1	84.07
	80	114.52	2.68	78.05
	75	120.69	3.475	71.18
	70	129.49	5.139	60.57
	65	144.63	8.999	46.22
	64	149.43	10.86	42.17
	63	154.03	12.526	36.05
	62	160.37	15.563	29.65
	61	167.96	17.3	22.24
	60	171.46	18.549	12.97
200	95	205.25	1.5	186.60
	90	211.83	2.04	175.68
	85	219.57	2.63	164.97
	80	229	3.35	153.44
	75	241.31	4.4	139.65
	70	258.86	6.43	122.83
	65	289.53	12.34	94.67
	64	299.63	14.95	89.95
	63	312.19	18.86	75.65
	62	327.88	25.22	96.55
	61	349.97	34.83	49.77
	60	376.59	48.06	28.92

Tortuosity should depend on the percolation fraction *p* and, near the percolation-threshold, should change by a critical exponent (see e.g. Ref.^10^), i.e. $\tau = A\left( {p - p_{c} } \right)^{u}$.

We consider $x_{0}$ = $\left\langle {l_{0} - L} \right\rangle$ to depend critically on $\left( {p - p_{c} } \right)$, and therefore assume:

2 $$x_{0} = a L\left( { p - p_{c} } \right)^{\beta }$$

**Figure 4:**
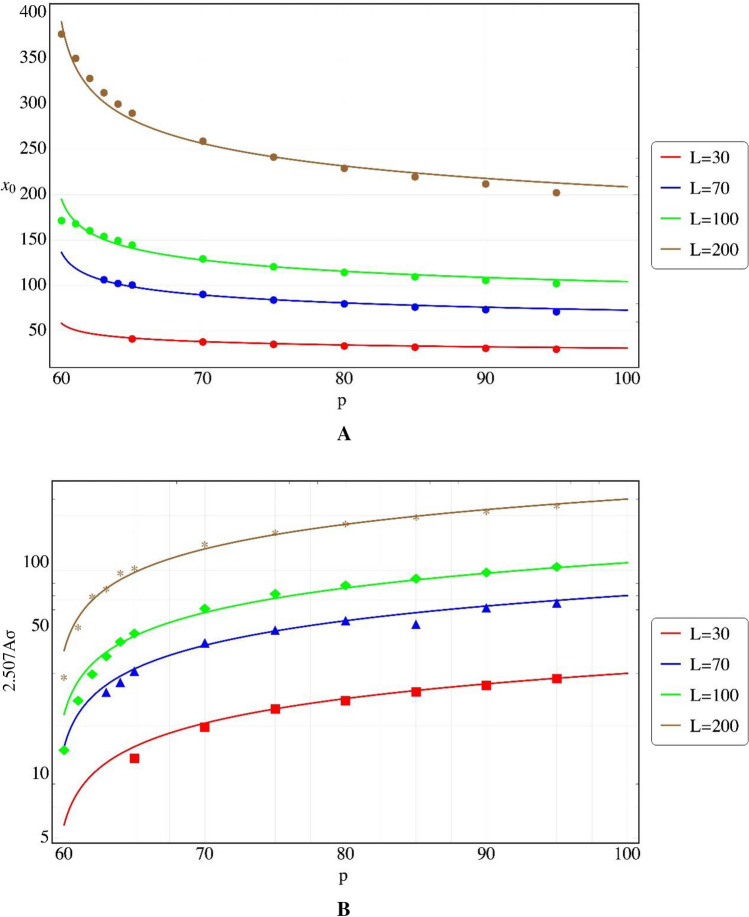
(Colors on line) Least square fits to Gaussian parameters of the output results near percolation threshold. (**A**) For $x_{0}$. (**B**) For *AT*. Critical behavior is observed.

In our numerical example, we ran a least square evaluation (Fig. 4A) of the results of the 1 → 2 → 0 case (Table 1) for $p_{c} = 59.3$ and values of *L* of 30, 70, 100 and 200, to get:

$$x_{0} \cong \left( {1.845 \pm 0.017} \right) L\left( {p - p_{c} } \right)^{ - 0.1538 \pm 0.005}$$

And $R^{2} = 0.9978.$ Or, for the tortuosity:

$$\tau = \left( {2.845 \pm 0.017} \right) \left( {p - p_{c} } \right)^{{ - \left( {0.1538 \pm 0.005} \right)}}$$

The average throughput (*AT*, the average number of the total active cells arriving at the end line) is estimated by the area under the Gaussian, given approximately by:$AT = 2.507A\sigma$ (Table 1). Near percolation the variable $AT$ also behaves critically, and using least square analysis of Table 1 values for the same variables as those used for $x_{0}$, we get (See Fig. 4B):

$$AT = \left( {0.222 \pm 0.006} \right) L\left( {p - p_{c} } \right)^{0.406 \pm 0.009} ,\;R^{2} = 0.9964.$$

## Discussion

The equality between the results of the SDP (1 → ... → *n* → 0, for $n \ge 1$) and the "forest-fire-like" systems for a *single wave* of AP (or fire) is not that surprising. A single wave in a heart like model of this type is really prevented from retracing. We expect, however, that under two or more waves, the systems would behave differently. For example, under multiple stimulations we expect no possibility of reentry in the forest fire model but a real possibility of a reentry mechanism for the SDP. Such stimulations will be addressed in future publications.

The "diagonal" case being considerably less constrained than the other two, has a much higher percolation threshold.

The question of front to back connecting routes and clustering, specifically around the percolating path, are not addressed here. They can be found in the FFM literature.

There exist several path-finding algorithms. For example, the A* or A-star algorithm (See e.g. Ref.^18^), which finds the best (shortest) route from a specific cell to a target cell, or a random walk algorithm (See e.g. Ref.^19^) which can also evaluate tortuosity. It would be interesting to compare near percolation routes of AP in the present model (e.g., the route in Fig. 1B) with these methods.

Figure 3B shows the Gaussian average outcome of the active cells arrival at the end row of the system. The emergence of a Gaussian distribution of the average transport seems natural since the inoperative cells are randomly spread and the average is governed by the central limit theorem. Table 1 provides the parameters of such average outcomes for different matrix sizes and *p*'s. The appearance of critical behaviors of both $x_{0}$ (and tortuosity, τ) and $AT$ (throughput) near percolation (Fig. 4), is similar to the hydrological^16^ or electrical results, although the critical exponents are different (e.g. for τ ~ − 0.15 vs. − 0.28 from Eq. 50 and values in Ref.^17^) and *L* does not have the same influence. We do not expect a precise correspondence since no "driving potential" exists here, as noted above.

Note that all following discussion pertain to the specific model treated here.

In the heart tissue, such percolation problems could arise when a portion of the heart tissue is riddled with scars^20^, fibrosis^20, 21^ or fatty globules^22^.

Fibrosis in the heart develops in many pathogeneses in the body (see the comprehensive Ref.^22^). It can induce a variety of heart problems by changing the tissue structure, such as fibrotic expansion of the cardiac interstitium, stiffer ventricles etc.^22^. We discuss here only arrhythmias, which can be prompted^23^ by fibrosis. As shown by Ref.^24^, results in rodent models imply that fibrotic areas cause slower kinetics or even a disruption of impulse propagation. These results are depicted in the present model by the tortuosity and transport on the one hand, and the approach to a complete AP stoppage for fibrosis percentage near percolation threshold on the other.

The relation between fibrosis and atrial fibrillation appears in several studies^25^. This and other fibrosis developed arrhythmic malfunctions are usually induced by an active reentry mechanism, a closed path on which an AP pulse is rotating, inducing target waves, spirals or double spirals in the tissue. Alongside with the fibrotic area in which a reentry route (RR) may develop, the "activation" of the RR needs additional factors. No activation can take place without a *unidirectional block* and the latter can probably be induced only by the influence of two or more consecutive AP waves arriving at the RR.

In the model discussed here, there can be no *active* reentry circuit for a movement of a *single AP front*. For, suppose an RR exists in the percolation matrix. In the absence of a unidirectional block, the development would be as follows. It is well known that when two AP waves meet, they annihilate each other. An AP reaching the RR on its way would enter and proceed by moving on it *in both forward directions*. Thus, if their passage times are different, the faster AP would emerge on the other end if it is open. In this case, the faster AP would also enter the route of the slower one and annihilate it. In any case, no circulating (active) reentry route would be possible.

The influence of a fibrotic spot (FS) in the heart tissue can depend on its position inside the heart. The delay and the lower AP amount reaching its other side caused by the FS, besides reducing contraction in the spot itself, can lead to problems in the adjoining tissue. If the FS encounters an obstacle, such as a scar, the heart boundary or a blood vessel, the region there may suffer too. If, however, the FS is surrounded by healthy tissue, then, under a single stimulation, the AP in the healthy tissue will arrive first and will in fact erase the delay of the FS in the next area. The scenario here is as follows. Consider an FS, of impaired tissue within a healthy one. The impairment can be a scar, fibrosis, tumor etc. We consider here only a fibrotic FS as treated here. If the action potential's conduction velocity (CV) is slowed down in FS, as we have shown it to be, one could assume that any action potential (AP) emerging from the FS, say AP1, would be delayed with respect to the AP in the healthy tissue outside A, say AP2. The former AP, AP1, could turn backwards without encountering AP2 creating an active reentry path. This however cannot happen without a unidirectional block. Thus, fortunately for animals, if the fibrotic area (FS) is surrounded by healthy tissue, the AP, moving faster in the healthy tissue than in the FS and arriving earlier on the other, distant, side of the FS, can move around the latter and squash (annihilate) any late arriving AP from the FS. The reason for this is the following. The AP2, being faster than AP1, can rotate around the distant boundary of FS and enter the FS itself, moving backwards there, and mutually annihilating with any AP1. In the present model this annihilation is carried out by "merging" of the two cells, i.e. by both activating the last intermediary cell between them, and the latter expiring, having refractory cells in both of its directions. Thus, if a healthy tissue surrounds the fibrotic one, it may quench the intermittent behavior of the FS. Otherwise, The FS can possibly be morbid for a fibrotic patient.

Although we have presented results for a normal distribution of the *average* behavior of *different* fibrotic tissues, for a specific patient it is a definite single fibrotic case with the erratic behavior shown as one of the individual graphs in Fig. 3A.

**Figure 5:**
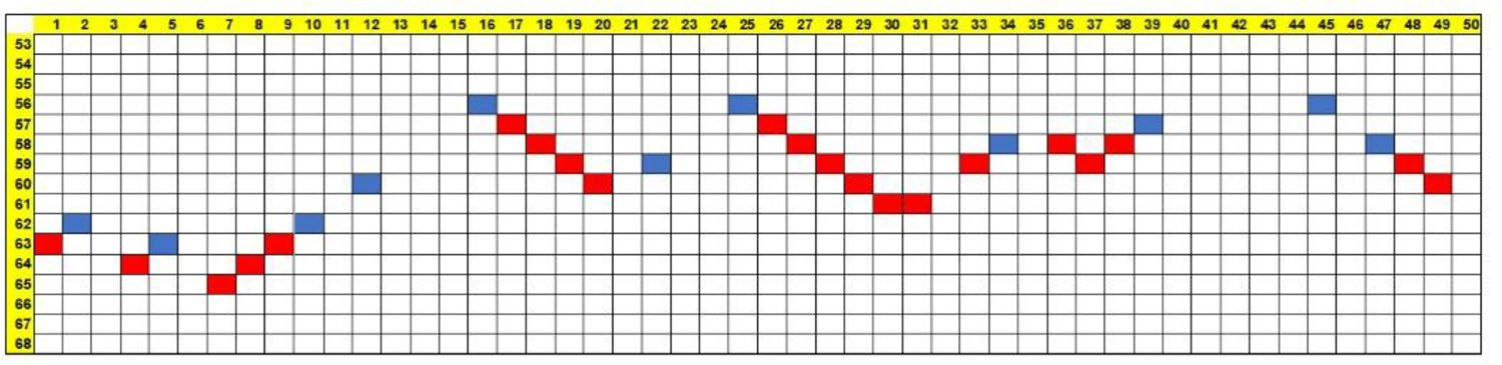
(Colors on line) Active (AP) cells arrivals at the end row of the matrix for a specific trial of a 50 × 50 fibrotic tissue (*p* = 75%). Abscissa – No. of cell along the row, Ordinate—Time of arrival (time units from wave starting time).

The spatial distribution of the arriving AP's is also of interest. In fibrotic tissues, the electricity emerges unevenly. There exist "breaking locations" where AP appear after its tortuous journey. Following breaking, these AP's can encounter inoperative cells on the last row of the matrix on both of their sides, in which case no additional cells become activated. If, however, one or both sides of the arriving AP were in the waiting (0) mode, there would be additional adjacent operative cells in the next generations. An example of this spatiotemporal behavior is shown in Fig. 5 for a 50 × 50 matrix with 25% fibrosis (*p* = 75).

It is seen that there are only 11 breaking locations (blue), in order of arrival: (A) cells Nos. 16, 25 and 41, arriving on generation 56. (B) location 39 arriving at 57 etc. Note that, for a specific patient, these locations would emit the AP intermittently in time at each incoming wave. The other 21 cells (red) are activated along the last row.

## Conclusions

The major results of this study for a fibrotic heart tissue are: (1) A fibrotic area induces delays in the active cells, reductions in their quantity and spreads in their arrival, both spatially and temporally. Both these delays and quantity reductions behave critically near percolation. **(**2) A single wave moving in a heart like model of a refractory period equal or larger than the active period, is identical to a movement in a forest fire model, including the percolation curves. This behavior can possibly be broken for multiple waves. (3) Therefore, even in a fibrotic area and even if surrounded by healthy setting, no active reentry mechanism can be initiated under a single stimulation. Conceivably, multiple waves or shorter refractory period, are needed for such a purpose.

## List of symbols

*L* (*n*): Matrix size (in cell numbers)
*p*: Percolation parameter (percentage of live cells of the total)
*q*: Percentage (of the total) of non-conducting cells = 100-p
*x*_0_: Gaussian model for the average time (in time units) when maximal number of AP's arrive at the end row.
*A*: Gaussian height, at x_0_
*σ*: Gaussian width (in time units)
0: Cell mode of "ready'
1,2: Cell modes of "refractory" (except 9)
9: "Non-conducting" cell mode
